# Supplementary material for: Disentangling the genetic basis of rhizosphere microbiome assembly in tomato
Source: Nat Commun. 2022 Jun 16;13:3228. doi: 10.1038/s41467-022-30849-9 (PMC9203511; doi:10.1038/s41467-022-30849-9)
Supplement: Supplementary file 1 — Supplementary Information [file 41467_2022_30849_MOESM1_ESM.pdf]

Disentangling the genetic basis of rhizosphere microbiome assembly in tomato

Oyserman *et al.*

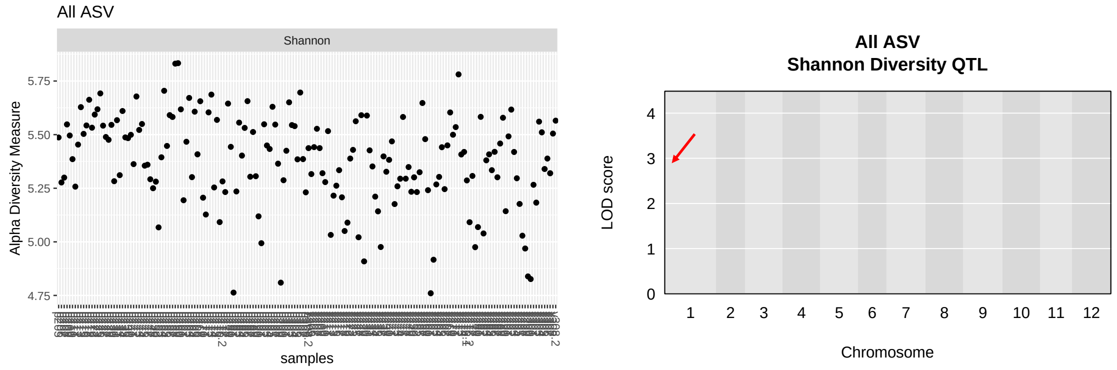

lodindex	lodcolumn	chr	pos	lod	ci_lo	ci_hi	asv	effect	heritability	allele
**1**	**Shannon_All**	**1**	**0.58584806**	**2.742001443**	**0.23412002**	**90.48919441**	**NA**	**-0.009596283**	**0.04091968**	**wild**
2	Shannon_CF	3	51.1168584	3.139555747	15.50220432	55.56201797	NA	-0.012594799	0.039807674	wild

Supplementary Figure 1. Shannon diversity QTLs using all ASV. A QTL analysis was conducted using Shannon diversity of each RIL as a quantitative trait. All ASV were included in this analysis.

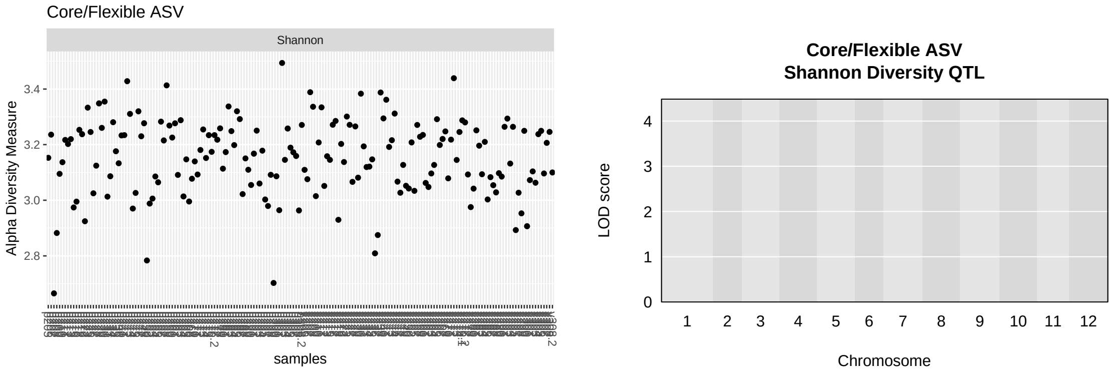

lodindex	lodcolumn	chr	pos	lod	ci_lo	[illegible]	[illegible]	[illegible]	[illegible]	[illegible]
1	Shannon_All	1	0.58584806	2.742001443	0.23412002	90.48919441	NA	-0.009596283	0.04091968	wild
**2**	**Shannon_CF**	**3**	**51.1168584**	**3.139555747**	**15.50220432**	**55.56201797**	**NA**	**-0.012594799**	**0.039807674**	**wild**

Supplementary Figure 2. Shannon diversity QTLs using Flexible/Core ASV. A QTL analysis was conducted using Shannon diversity of each RIL as a quantitative trait. Only rhizosphere enriched ASV found in 50% or more RIL accessions were included.

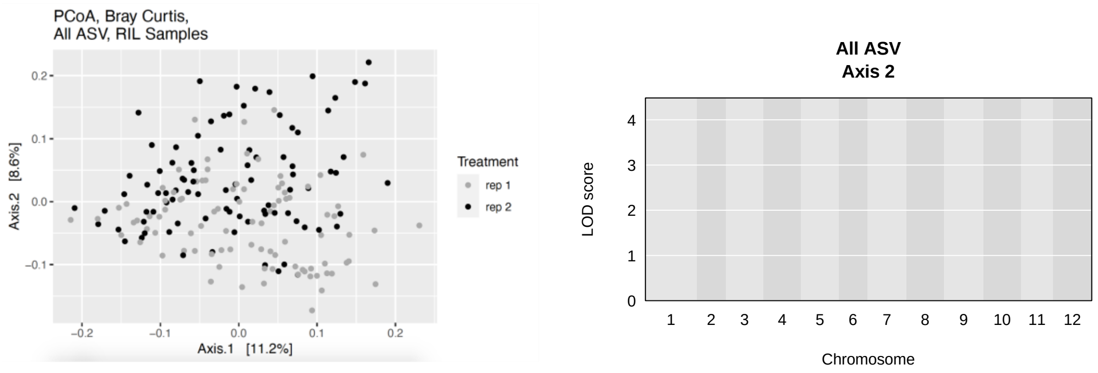

lodindex	lodcolumn	chr	pos	lod	ci_lo	ci_hi	asv	effect	heritability	allele
2	allAxis2	6	38.4400203	3.27968774	36.282317	44.957515	NA	-0.1230122	0.15832824	wild

Supplementary Figure 3. PCoA QTLs. A QTL analysis was conducted using PCoA axis 1 and 2 of each RIL as a quantitative trait. All ASV were included.

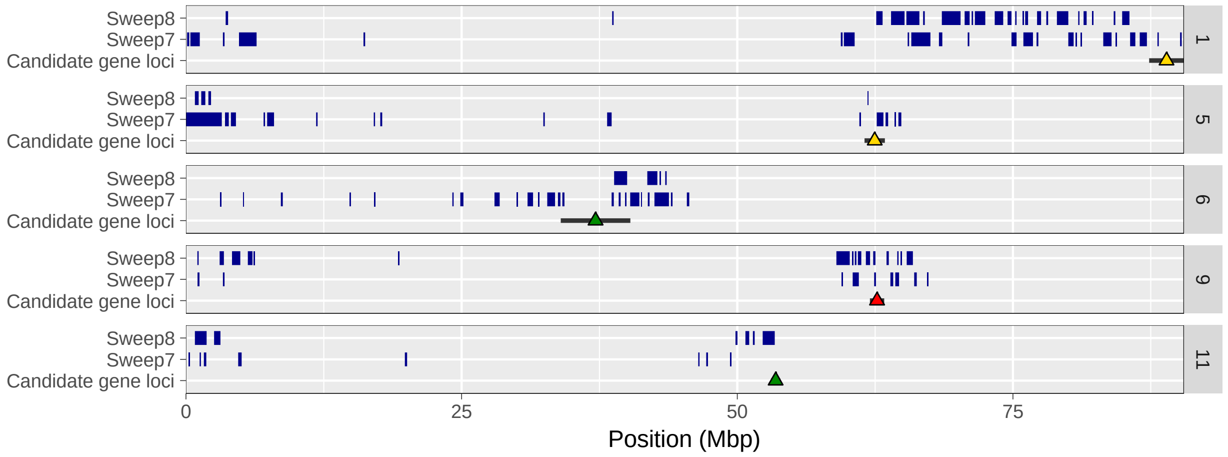

Supplementary Figure 4. Overlay of gene sweeps on QTL positions. The distribution of gene sweeps linked to the initial domestication and improvement for fruit (quality) traits (sweep7 and sweep8 respectively)31 overlaid with the prioritized QTLs on chromosomes 1, 5, 6, 9, 11.

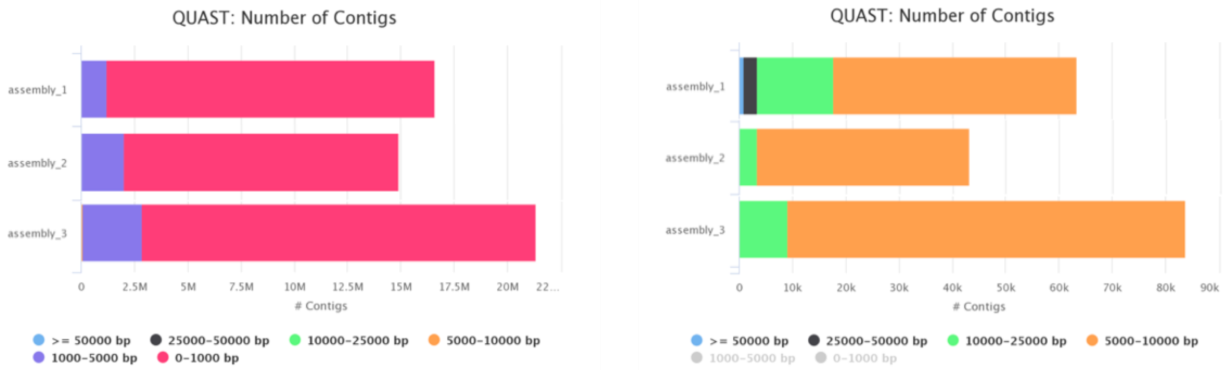

Supplementary Figure 5. MultiQC Contigs across different assembly strategies. Bar plots generated by MultiQC showing the number of contigs with different ranges of length in the metagenomic assemblies. Figure S1A provides an overview of all the contigs and large contigs are focused in Figure S1B. The bars are color coded based on the length of contigs. "assembly_1", "assembly_2" and "assembly_3" indicated the first, second and third assembly respectively. The third assembly yielded the greatest total number of contigs but most large contigs (≥ 10 Kbp) were successfully assembled in the first assembly.

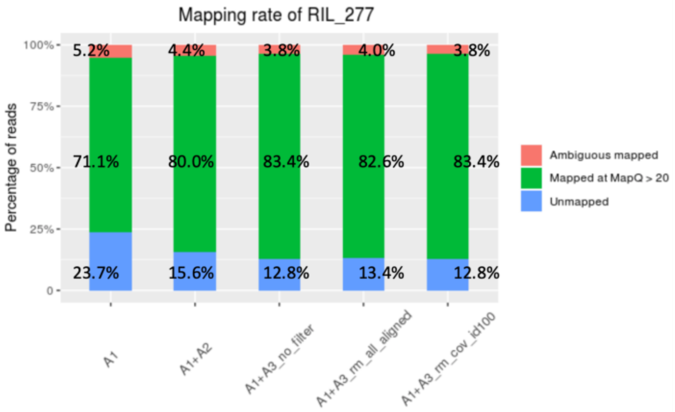

Supplementary Figure 6. Metagenomic depth of randomly sampled RIL. Mapping rates of RIL 277 in the benchmarking test on the filtering sensitivity of overlapping contigs. A1: the first assembly using reads from 11 parental (6 modern, 5 wild) and 1 bulk-soil samples; A2: The reads from the RIL metagenomes were mapped to assembly A1, and all unmapped reads were assembled; A3: Again, as with assembly A2, the third assembly used unmapped reads, but also included ambiguously mapped and low-quality mapped (MapQ < 20) reads from RIL samples. A1 and A2 were merged directly because there were no overlapping contigs, which was represented by "A1+A2" in the figure. The filtering of overlapping contigs in A3 was divided to 3 levels of stringencies: removing all aligned contigs (the most stringent), removing the overlapping contigs with 100% identity and coverage, and keeping all the aligned contigs (the loosest), which were represented by "A1+A3_rm_all_aligned", "A1+A3_rm_cov_id100" and "A1+A3_no_filter" respectively in the figure.

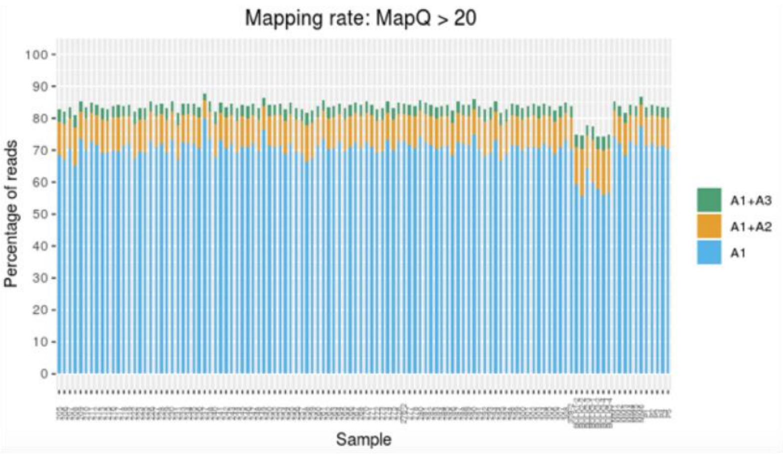

Supplementary Figure 7. Metagenomic depth of all samples. The mapping rates for three metagenomic assemblies. The first assembly and two merged assemblies were indexed and treated as the reference respectively in the backmapping for the metagenomic reads. For each sample, the number of reads with a mapping quality equal or greater than 20 were counted by using SAMtools and divided by the total number of reads per sample (including both reverse and forward reads). This figure shows that compared to the first assembly, the read recruitment for the merged assemblies were improved by adding unmapped reads, ambiguously mapped reads, and mapped reads with a low mapping quality score (MapQ < 20) from the RIL accessions. The mapping rates for the final assembly (A1+A3) were from 75% to 88%.

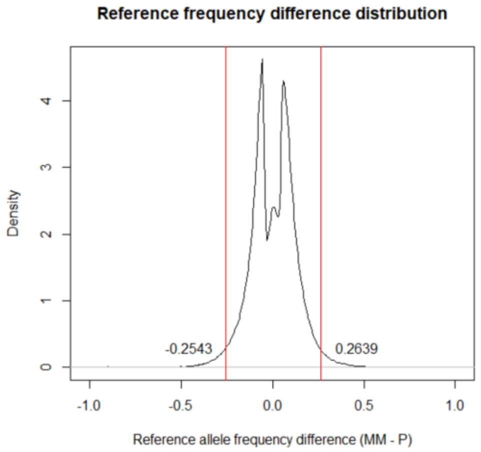

Supplementary Figure 8. SNV feature selection. Distribution of difference in SNP reference allele frequency between the MM and P metagenomes (MM – P). Red lines indicate the 95% CI and the corresponding values indicate the significance thresholds. The 30,932 SNPs with reference allele frequency difference outside the 95% CI are used for further selection. The two peaks just around 0 arise from the addition of the SNPs that are called by inStrain in one dataset, but not in the other, and are thus assumed to comprise 100% reference alleles. This often leads to SNPs being recognized in one dataset with at most 95% reference allele frequency (5% SNP frequency is minimum requirement), while in the other dataset there is 100% SNP identity, resulting in the large peaks of barely different SNP loci. The SNPs in between are a result of identical reference allele frequencies, but this need not indicate similar variant alleles.

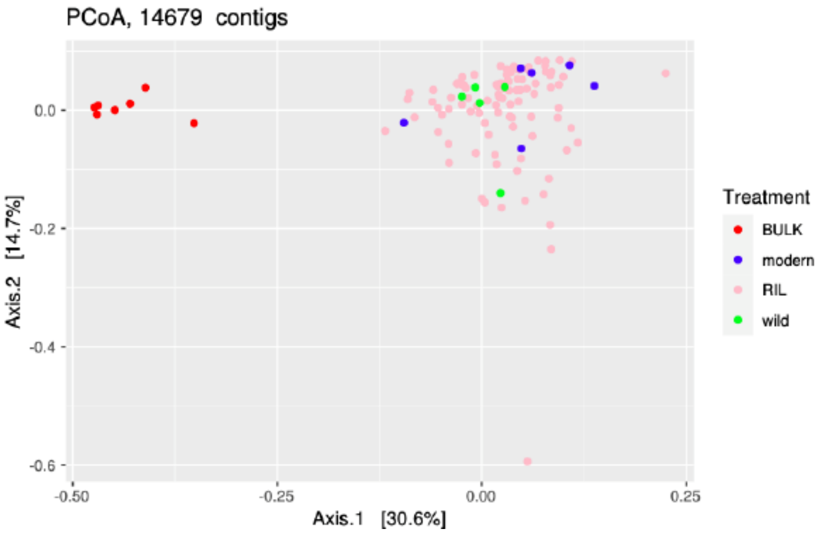

Supplementary Figure 9. Principal coordinate analysis (PCoA) using Bray's distances showed that metagenome contigs were separated between the group of bulk soil and rhizosphere.
