# Supplementary material for: Disentangling the genetic basis of rhizosphere microbiome assembly in tomato
Source: Nat Commun. 2022 Jun 16;13:3228. doi: 10.1038/s41467-022-30849-9 (PMC9203511; doi:10.1038/s41467-022-30849-9)
Supplement: Supplementary file 3 — Description of Additional Supplementary Files [file 41467_2022_30849_MOESM3_ESM.pdf]

Description of Additional Supplementary Files

File Name: Supplementary Data 1
Description: Various meta data collected for each pot harvested. Includes the unique ID, the replicate (Color coded), the germination day and date, as well as the days of growth. Furthermore, this metadata sheet also includes the date of harvest number, the number of leaves at harvest (Leaves), the mass of the rhizosphere harvested in grams (Rhizosphere), the mass of the bulk soil remaining in grams (Soil_Before), and the plant dry weight in grams (PDW).

File Name: Supplementary Data 2
Description: The metadata on each ASV including its raw abundance in each sample. The first columns are the kingdom, phylum, class, order, family, genus, species. The remaining columns represent the sample IDs.

File Name: Supplementary Data 3
Description: The abundance of ASVs after CSS normalization and filtering with each column representing a different sample.

File Name: Supplementary Data 4
Description: Some basic statistics about each ASV, including the number of zeros within the RIL population (zeroes), the number of non-zero abundances (nz), the mean (mean), the mean when excluding zero counts (nz_mean), the sum, minimum (min), maximum (max), the normalized standard deviation (norm_sd), the normalized standard deviation of non-zero counts (norm_sd_nz) and finally the a classification into either core, flexible or uncommon (ecology).

File Name: Supplementary Data 5
Description: A fasta file for the ASVs identified in samples harvested on 5/19/2019.

File Name: Supplementary Data 6
Description: The ASV QTLs identified including the ASV, the chromosome (chr) and position in Mbps (pos), the LOD score (lod), the low (ci_lo) and high (ci_hi) 95% confidence intervals in Mbps, the effect, heritability, whether a wild or modern allele was observed at that position. In addition, taxonomical information is included.

File Name: Supplementary Data 7
Description: The taxonomy output and quality assessments of CheckM of the metagenome assembled bins

File Name: Supplementary Data 8
Description: The taxonomy output from Kraken on the contigs 10kb and greater in size.

File Name: Supplementary Data 9
Description: The bin QTLs identified including the bin id, the chromosome (chr) and position in Mbps (pos), the LOD score (lod), the low (ci_lo) and high (ci_hi) 95% confidence intervals in Mbps, the effect, heritability, whether a wild or modern allele was observed at that position.

File Name: Supplementary Data 10

Description: The contig QTLs identified including the contig id, the chromosome (chr) and position in Mbps (pos), the LOD score (lod), the low (ci_lo) and high (ci_hi) 95% confidence intervals in Mbps, the effect, heritability, whether a wild or modern allele was observed at that position. In addition, taxonomical information is included.

File Name: Supplementary Data 11
Description: A table summarizing the output from the independent population of RIL accessions. The metadata includes the sample unique identifier (Replicate), whether a sample was a bulk soil, parental (Solanum lycopersicum cv Moneymaker, M; Solanum pimpinellifolium, P), the RIL accession number (three digit code), or bulk soil (Bulk). In the next three columns, the SNP information for markers 2274 linked to Streptomyces, and markers 464 and 3142 linked to Cellvibrio (A allele – Solanum lycopersicum cv Moneymaker, B allele - Solanum pimpinellifolium). Finally, the last two columns are the Streptomyces (ASV3) and Cellvibrio (ASV9) CSS normalized abundances. The ASV numbering is different as this was an independent experiment.

File Name: Supplementary Data 12
Description: A summary of the five prioritized QTL regions. In the first sheet 'Info', key information about each QTL is summarized including a unique ID (QTL_ID), the left and right side of the confidence interval as well as its size, genus and number (and type) of combined QTLs to form this prioritized region are provided. In subsequent sheets, the positions, annotations, expression patterns and gene sweeps are provided.

File Name: Supplementary Data 13
Description: A summary of the five prioritized QTL regions in a single sheet and with additional calculations including the log2 of the leaf/root ratio. An arbitrarily small value (0.1) was added to both root and shoot calculations to prevent fold calculations approaching negative infinity.

File Name: Supplementary Data 14
Description: Focused annotations for the contigs with QTLs including, HMMER, Hotpep, DIAMOND as well as a unique gene ID.

File Name: Supplementary Data 15
Description: Annotations for all contigs that were 10 kb and greater.

File Name: Supplementary Data 16
Description: Summary of output from AntiSmash.

File Name: Supplementary Data 17
Description: The SNV QTLs identified including the SNV id, the chromosome (chr) and position in Mbps (pos), the LOD score (lod), the low (ci_lo) and high (ci_hi) 95% confidence intervals in Mbps, the effect, heritability, whether a wild or modern allele was observed at that position. In addition, taxonomical information is included, as well as annotation.

File Name: Supplementary Data 18
Description: Assembly statistics from the three assemblies and their merged results.

File Name: Supplementary Data 19
Description: Determining which contig features were selected

File Name: Supplementary Data 20
Description: MMseqs results at 50% and 70% protein ID cutoffs.

File Name: Supplementary Data 21
Description: Shannon diversity for all RIL accessions.

File Name: Supplementary Data 22
Description: PCoA axis 1 and 2 for all RIL accessions.
