# Supplementary material for: Disentangling the genetic basis of rhizosphere microbiome assembly in tomato
Source: Nat Commun. 2022 Jun 16;13:3228. doi: 10.1038/s41467-022-30849-9 (PMC9203511; doi:10.1038/s41467-022-30849-9)
Supplement: Supplementary file 26 — Reporting Summary [file 41467_2022_30849_MOESM26_ESM.pdf]

nature portfolio

Corresponding author(s): Ben O Oyserman
Jos M Raaijmakers

Last updated by author(s): 05/19/2022

Reporting Summary

Nature Portfolio wishes to improve the reproducibility of the work that we publish. This form provides structure for consistency and transparency in reporting. For further information on Nature Portfolio policies, see our Editorial Policies and the Editorial Policy Checklist.

Statistics

For all statistical analyses, confirm that the following items are present in the figure legend, table legend, main text, or Methods section.

n/a	Confirmed	
☐	☒	The exact sample size (*n*) for each experimental group/condition, given as a discrete number and unit of measurement
☐	☒	A statement on whether measurements were taken from distinct samples or whether the same sample was measured repeatedly
☐	☒	The statistical test(s) used AND whether they are one- or two-sided<br>*Only common tests should be described solely by name; describe more complex techniques in the Methods section.*
☐	☒	A description of all covariates tested
☐	☒	A description of any assumptions or corrections, such as tests of normality and adjustment for multiple comparisons
☐	☒	A full description of the statistical parameters including central tendency (e.g. means) or other basic estimates (e.g. regression coefficient) AND variation (e.g. standard deviation) or associated estimates of uncertainty (e.g. confidence intervals)
☐	☒	For null hypothesis testing, the test statistic (e.g. *F*, *t*, *r*) with confidence intervals, effect sizes, degrees of freedom and *P* value noted<br>*Give P values as exact values whenever suitable.*
☒	☐	For Bayesian analysis, information on the choice of priors and Markov chain Monte Carlo settings
☒	☐	For hierarchical and complex designs, identification of the appropriate level for tests and full reporting of outcomes
☒	☐	Estimates of effect sizes (e.g. Cohen's *d*, Pearson's *r*), indicating how they were calculated

Our web collection on statistics for biologists contains articles on many of the points above.

Software and code

Policy information about availability of computer code

Data collection	no software was used in data collection.
Data analysis	bedtools genomecov version: 2.29.2, CheckM version: 1.1.1, DRAM v1.2, Flexbar version 2.5, InStrain version: 1.4.0, Kraken2 version: 2.0.8, Limma version 3.22.7, MEGAHIT version 1.2.9, Metabat2 version 2:2.15, metagenomeSeq version 1.28.2, MMseqs2 Version: 13.45111 , MultiQC version 1.8, MUMmer version: 4.0.0, PANDASeq Assembler version 2.11, Prodigal version: 2.6.3, Prokka version: 1.14.6, python3, R Statistical Software 4.0.1, SAMtools version 1.10, SPAdes version 3.13.2. Code is available at Zenodo https://doi.org/10.5281/zenodo.6561541

For manuscripts utilizing custom algorithms or software that are central to the research but not yet described in published literature, software must be made available to editors and reviewers. We strongly encourage code deposition in a community repository (e.g. GitHub). See the Nature Portfolio guidelines for submitting code & software for further information.

Data

Policy information about availability of data

All manuscripts must include a data availability statement. This statement should provide the following information, where applicable:

- Accession codes, unique identifiers, or web links for publicly available datasets
- A description of any restrictions on data availability
- For clinical datasets or third party data, please ensure that the statement adheres to our policy

The sequencing data generated in this study are available under BioProject ID PRJNA787039677 (16S amplicons) and PRJNA789467 (shotgun metagenomics). The Silva database used for taxonomic classification is available at https://www.arb-silva.de/. Metagenome assembled genomes are available at https://doi.org/10.5281/zenodo.6561541. Additional databases used include Silva (https://www.arb-silva.de/), UniRef (https://www.uniprot.org/), Pfam (https://pfam.xfam.org/), dbCAN (https://bcb.unl.edu/dbCAN2/) and KEGG (https://www.kegg.jp/). A custom database was used for Kraken annotation and, due to size limitations, is available upon

request.

Field-specific reporting

Please select the one below that is the best fit for your research. If you are not sure, read the appropriate sections before making your selection.

☐ Life sciences ☐ Behavioural & social sciences ☒ Ecological, evolutionary & environmental sciences

For a reference copy of the document with all sections, see nature.com/documents/nr-reporting-summary-flat.pdf

Ecological, evolutionary & environmental sciences study design

All studies must disclose on these points even when the disclosure is negative.

Study description	We investigated the microbiome of approximately 100 different genotypes of a recombinant inbred line of tomatoes. For each genotype, 16S sequencing was conducted with biological replicates (e.g. individual plants were samples). For the shotgun metagenome sequencing, a single replicate was sequenced. In addition, second experiment was conducted with the same recombinant inbred line as validation. In this validation experiment, between 4-5 biological replicates of each genotype was sampled. Exact replicate numbers are reported in the supplemental information.
Research sample	In this study, a research sample was the rhizosphere of a tomato from a genetically diverse recombinant inbred line crossed between Solanum lycopersicum var Moneymaker and Solanum pimpinellifolium. The rational to use this population is because it represents the genetic diversity found in modern and wild tomato and allows us to understand how domestication may have impacted microbiome assembly on a genetic level. The plants were grown in greenhouses at Netherlands Institute of Ecology and seeds were provided by co-author WL.
Sampling strategy	The sample size was determined based on the availability of the recombinant inbred line. All possible genotypes in this population were included. To sample the rhizosphere, plants were taken from pots and only the soil that was tightly adhered to the roots after shaking was sampled for DNA extraction and sequencing.
Data collection	The metadata on each plant was recorded by a coauthors BOO, SSF and VC by pen and paper or directly into excel. The plants were grown in a greenhouse and monitored. Various plant phenotypes were collected before the sampling of the rhizosphere. Data on the microbiome from the rhizosphere was collected by amplicon and shotgun sequencing.
Timing and spatial scale	The rhizosphere samples were collected throughout the day on April 18, 19, and 20 of 2019. Sampling was done on numerous days to accommodate the number of plants that needed to be sampled. The spatial scale was such that there was a single plant per pot (300g soil).
Data exclusions	no data was excluded from the analysis.
Reproducibility	One attempt was made to replicate the results using an independent bulk segregation study with 16S amplicon sequencing. This validation was successful.
Randomization	All plants were randomized within the greenhouse. Furthermore, because of spatial inconsistencies that occur within a greenhouse, all plants were randomly rotated throughout the greenhouse approximately twice per week. Various covariates such as collection date were used in the QTL analysis as covariates.
Blinding	blinding was not conducted and was not relevant to the study as there was no experimental manipulation and the investigators had no knowledge of the genetic background of the recombinant inbred line population.

Did the study involve field work? ☐ Yes ☒ No

Reporting for specific materials, systems and methods

We require information from authors about some types of materials, experimental systems and methods used in many studies. Here, indicate whether each material, system or method listed is relevant to your study. If you are not sure if a list item applies to your research, read the appropriate section before selecting a response.

Materials & experimental systems

n/a	Involved in the study	
☒	☐	Antibodies
☒	☐	Eukaryotic cell lines
☒	☐	Palaeontology and archaeology
☒	☐	Animals and other organisms
☒	☐	Human research participants
☒	☐	Clinical data
☒	☐	Dual use research of concern

Methods

n/a	Involved in the study	
☒	☐	ChIP-seq
☒	☐	Flow cytometry
☒	☐	MRI-based neuroimaging
